# Can teaching agenda-setting skills to physicians improve clinical interaction quality? A controlled intervention

**DOI:** 10.1186/1472-6920-8-3

**Published:** 2008-01-14

**Authors:** Hector P Rodriguez, Michael P Anastario, Richard M Frankel, Esosa G Odigie, William H Rogers, Ted von Glahn, Dana G Safran

**Affiliations:** 1 Department of Health Services, School of Public Health and Community Medicine, University of Washington, Seattle, WA, USA; 2 Uniformed Services University of the Health Sciences, F. Edward Hébert School of Medicine, Bethesda, MD, USA; 3 The Regenstrief Institute, Indiana University School of Medicine, Indianapolis, IN, USA; 4 The Health Institute, Institute for Clinical Research and Health Policy Studies, Tufts-New England Medical Center, Boston, MA, USA; 5 Department of Medicine, Tufts University School of Medicine, Boston, MA, USA; 6 Pacific Business Group on Health, San Francisco, CA, USA

## Abstract

**Background:**

Physicians and medical educators have repeatedly acknowledged the inadequacy of communication skills training in the medical school curriculum and opportunities to improve these skills in practice. This study of a controlled intervention evaluates the effect of teaching practicing physicians the skill of "agenda-setting" on patients' experiences with care. The agenda-setting intervention aimed to engage clinicians in the practice of initiating patient encounters by eliciting the full set of concerns from the patient's perspective and using that information to prioritize and negotiate which clinical issues should most appropriately be dealt with and which (if any) should be deferred to a subsequent visit.

**Methods:**

Ten physicians from a large physician organization in California with baseline patient survey scores below the statewide 25th percentile participated in the agenda-setting intervention. Eleven physicians matched on baseline scores, geography, specialty, and practice size were selected as controls. Changes in survey summary scores from pre- and post-intervention surveys were compared between the two groups. Multilevel regression models that accounted for the clustering of patients within physicians and controlled for respondent characteristics were used to examine the effect of the intervention on survey scale scores.

**Results:**

There was statistically significant improvement in intervention physicians' ability to "explain things in a way that was easy to understand" (p = 0.02) and marginally significant improvement in the overall quality of physician-patient interactions (p = 0.08) compared to control group physicians. Changes in patients' experiences with organizational access, care coordination, and office staff interactions did not differ by experimental group.

**Conclusion:**

A simple and modest behavioral training for practicing physicians has potential to positively affect physician-patient relationship interaction quality. It will be important to evaluate the effect of more extensive trainings, including those that work with physicians on a broader set of communication techniques.

## Background

Physicians and medical educators have repeatedly acknowledged the inadequacy of communication skills training in the medical school curriculum and opportunities to improve these skills in practice [1-6]. Patient-centered communication, however, is no longer a boutique concept and is increasingly seen as critical for care outcomes that rely on patient engagement. For example, evidence suggests that effective physician communication can improve patients' adherence to treatment recommendations [7,8] and is strongly associated with patient loyalty to physicians [9-11]. Moreover, reports reveal that the quality of physician-patient relationships has been declining in recent years [12,13], highlighting the need for improvement.

Several studies have demonstrated the feasibility of bringing vital skills for effective communication to large numbers of practicing clinicians [14,15], but there continues to be a dearth of evidence that communication interventions significantly improve physician communication or the quality of the physician-patient relationships. Previous studies are limited in many ways. First, studies have failed to use control groups [16-19], pre-intervention [20-23] or follow-up patient assessments [15,16,20] thereby significantly limiting causal inference. Second, studies generally have not evaluated a random cross-section of active patients in physician practices and those that have, use generalized measures limited to a single aspect of physician-patient relationships [24]. Third, some studies have relied on training programs with extensive time-commitments that may be impractical for physicians in busy practices [20]. This study evaluates the effects of a short face-to-face communication training program followed by two group teleconferences on the quality of physician-patient relationships using a random cross-section of patients in the participating physicians' practices.

Our study contributes to the literature in important ways. First, the intervention includes physicians in the same group practice who were exposed to the intervention collectively. Other communication interventions have involved physicians who ultimately return to their practices with other physicians who did not receive similar training. Changing the context and conversation at the practice has the potential to be a powerful training reinforcement. Second, this study uses a well-validated, widely used survey that assesses multiple dimensions of patients' experiences with care, including assessments of interaction quality, care coordination, and willingness to recommend the physician. Assessing multiple dimensions of physician-patient relationships can help clarify which aspects are most strongly affected by physician agenda-setting training. Third, we adapted a skill from the Four Habits Model of Clinician Patient Communication ("Four Habits Model"), a validated education and research framework that has been used successfully in large and small group practices [14,25]. Finally, our sample includes physicians exposed to pay-for-performance incentives targeted at improving the quality of physician-patient relationships. As a result, physicians in both the intervention and control groups have considerable motivation to improve. Thus, we are able to evaluate the extent to which the communication intervention contributes to improvement beyond what is achieved by physicians who have motivation (pay for performance incentives) and performance information (reports), but who do not receive training or assistance with improvement.

## Methods

### Physician sampling

Study participants were physicians from a large multispecialty physician organization in California, United States. Using baseline patient survey results, the medical group identified a set of lower performing medical practices, including several solo specialist practices. Target practices were comprised of physicians at or below the statewide 25^th ^percentile of performance. Most physicians were invited through a discussion with the medical group administrator and the physician practice site leader. Of four primary care practice sites that were invited to participate in the intervention, two sites agreed to participate and two declined. Of four specialty care practices (solo and shared) invited to participate, three practices agreed to participate and one declined. The most common reason for declining participation was concern about work burden due to staffing changes and appointment supply constraints. Ten physicians from three practice sites ultimately participated in the intervention. Of the 10 intervention group physicians, 7 were adult Primary Care Physicians from a single practice, 2 were Orthopedic surgeons in a shared practice, and one was an Ophthalmologist in solo practice. Eleven physicians from three different practice sites with patient survey scale scores below the 25th percentile were selected as matched controls based on survey scores, geography, specialty, and practice size. Prior to the intervention, all physicians (intervention and controls) received feedback about their patient survey results in one-on-one sessions with their physician site leader. Performance feedback reports highlighted the physician's performance relative to the medical group's physicians in the same specialty and to the statewide 90^th ^percentile of performance.

### The Intervention

The communications training intervention consisted of a 3-hour evening workshop (19 October 2005) and two 45-minute teleconference calls conducted 3 and 7 weeks after the workshop. The evening program provided "agenda-setting" skills as a means of initiating and conducting patient office visits. The 3-hour program consisted of three segments: 1) didactic presentation, 2) video-clips of clinical encounters followed by full-group discussion, and 3) small group exercises, including role-plays of scripted clinical cases with feedback to the clinician "actor" (the intervention participant) and group discussion. The concept of "agenda-setting" that was the foundation for the workshop was adapted from "Habit-#1" in the Four Habits Model. The "agenda-setting" workshop aimed to engage clinicians in the practice of initiating patient encounters by eliciting the full set of concerns from the patient's perspective and using that information to prioritize and negotiate which clinical issues should most appropriately be dealt with and which (if any) should be deferred to a subsequent visit. This approach stands in sharp contrast to the more common practice of focusing on the first clinical issue named by the patient and asking, as the visit concludes, whether there is "anything else" that patient hoped to cover.

Three intended benefits of "front-loading" the process of eliciting the patients' list of concerns are (i) to afford the clinician more complete information with which to plan the encounter, (ii) to focus the encounter around the priorities the patient assigns to his or her concerns (i.e., being patient or relationship centered), and (iii) to identify important clinical concerns that may not have been part of the patient's stated reason for the visit. Communications researchers have repeatedly found that patients' first stated concern is not necessarily the chief concern from their perspective [26,27].

At 3 and 7 weeks post-intervention (November 10^th ^and December 8^th^, 2005), participants were asked to participate in a 45-minute group teleconference to discuss their experiences of employing the focal skills from the intervention in their practices. Participants were asked to prepare for the call by keeping notes about encounters where they noticed particular successes or challenges related to using the agenda-setting strategies with their patients. Calls were facilitated by the workshop instructors (RMF, DGS), who made an effort to ensure that each clinician participant reported on at least one experience during each call. Clinicians were also encouraged to coach one another and suggest strategies when colleagues reported about challenging aspects of an encounter.

### Study questionnaire

The study questionnaire was an abbreviated version of the Ambulatory Care Experiences Survey (ACES) [28], a validated survey that measures patients' experiences with a specific, named physician and that physician's practice. For this study, 4 summary measures of patients' experiences were assessed: quality of physician-patient interaction (k = 5), organizational access (k = 3), care coordination (k = 2), and office staff interactions (k = 1). The ACES questions and composite measures achieve physician-level reliability exceeding 0.70 with samples of 45 patients per physician [28,29]. As detailed elsewhere [28], Ambulatory Care Experiences Survey (ACES) summary scores range from 0 to 100 points, with higher scores indicating more favorable performance. Summary scores are computed for each respondent based on the unweighted average of responses to all items comprising the measure.

### Patient sampling

Baseline surveys were administered in April 2005 to a random sample of approximately 100 commercially-insured patients per physician who visited their physician between January-December 2004. A second survey was administered in March 2006 to a random sample of approximately 160 commercially-insured and Medicare-insured patients per physician who visited their physician between August-December 2005. Finally, a third survey was administered in March 2007 to a random sample of approximately 100 commercially-insured patients per physician who visited their physician from April-December 2006.

### Analyses

A total of 2,949 completed surveys were received for an overall response rate of 39.6%. The final analytic sample included 2,081 patients from 21 physicians (average patients per MD = 96.1). To address differences in the patient sampling criteria across baseline and follow-up survey administration efforts (e.g., commercially-insured only vs. commercially-insured plus Medicare-insured), the analytic sample excluded patients over 65 years of age (n = 640). Respondents who did not confirm the named physician as their physician and/or reported no visits with the physician during the previous 12 months (n = 228) were also excluded from the analytic sample.

The pre-intervention patient sample from the 2005 survey administration ("baseline 1") included 651 patients across the 21 physicians (intervention and controls). Administrative data including detailed visit information from August 2005-April 2006 was collected for all patients surveyed during the second survey administration effort. These data were used to establish the date of the patients' last physician visit before completing the survey. Patients whose last physician visit prior to completing the survey occurred before the intervention (August 1 – Oct 19, 2005) were considered part of the pre-intervention sample ("baseline 2"; n = 327). As a result, the overall pre-intervention patient sample includes 978 unique patients. Patients whose last physician visit prior to the survey occurred after the intervention (Oct 19, 2005-April 30, 2006) were considered part of the post-intervention sample (n = 515). These responses were grouped with patients from the 2007 survey administration effort (n = 588), yielding a total of 1,103 patients in the post-intervention group.

Socio-demographic characteristics were compared between the patients of control (n = 1,187) and intervention (n = 894) physicians. For this descriptive analysis, t-tests were used to compare continuous variables and χ^2 ^tests were used to compare dichotomous and categorical variables.

Multilevel regression models were used to examine the effect of the intervention on ACES summary scores. These analyses used the XTMIXED module in STATA 9.2 to take account for clustering of respondents within physicians using random effects. We included patient age, gender, education, race, self-rated physical health, sample derivation (to distinguish "baseline 1" from "baseline 2"), and physician-patient relationship duration as covariates in all regression models. In order to test whether the intervention had an effect on the ACES summary measures and items, two-way interactions between study group status (intervention group vs. control group) and the time of patients' most recent experiences with the physician relative to the intervention (pre-intervention vs. post-intervention) were tested.

Participation in the follow-up teleconferences was fairly high. Of the 10 physicians, 7 participated in both teleconferences, 1 participated in the first teleconference only, and 2 did not participate in either call. Physicians were asked to jot down notes about the benefits and challenges of incorporating the agenda-setting techniques into practice. During each of the follow-up teleconferences, physicians shared their experiences, discussed opportunities to improve the implementation of the newly-acquired skills, and provided one another with social support. Detailed notes were taken by one of the workshop instructors during each of the follow-up teleconferences. These notes were then transcribed and analyzed in order to identify recurring themes and perspectives.

## Results

### Patient Survey

Respondent characteristics differed between patients of physicians in the control and intervention groups (Table 1). Compared to patients of control group physicians, patients of intervention group physicians were more likely to have graduated college (50% vs. 25%, p < 0.001), to be non-white (p < 0.001), and had shorter-term relationships with the named physician (p < 0.001). Differences in respondent characteristics supported the inclusion of these covariates in multilevel regression models.

**Table 1:** Survey Respondent Characteristics, by Experimental Group

Patient Characteristics	Control Physicians	Intervention Physicians	
	(n = 1,187)	(n = 894)	
Average # patients/MD	107.9 (21.3)	89.4 (24.2)	
Age	48.5 (10.9)	49.8 (10.3)	**
Gender (% male)	44%	39%	*
			
Education			***
Less than high school	3.2%	6.1%	
High school graduate	73.4%	31.6%	
College graduate & beyond	23.4%	67.8%	
			
Race			
White	77%	61%	***
Black	2%	11%	***
Hispanic	7%	5%	
Asian	4%	17%	***
Other	14%	9%	***
			
Self Rated Physical Health	56.1 (25.2)	57.3 (27.4)	
			
Physician-Patient Relationship Duration			***
< 6 months	7%	10%	
6 months – 1 year	9%	14%	
1–2 years	17%	28%	
3–5 years	17%	23%	
> 5 years	50%	25%	
			
Number of Chronic Conditions	1.6 (1.5)	1.5 (1.4)	

*p < 0.05, **p < 0.01, ***p < 0.001

In adjusted analyses, the quality of physician-patient interaction scale scores of intervention physicians improved by 2.9 points after the intervention (from 85.8 to 88.7). This difference was marginally significant (p = 0.08) compared to changes observed among the control group physicians, which remained relatively stable over the study period (range: 85.6–85.9) (Table 2). There was statistically significant improvement in intervention physicians' ability to "explain things in a way that was easy to understand" compared to physicians in the control group (p = 0.02). Similarly, there were marginally significant improvements in intervention physician patients' willingness to recommend them to family or friends (p = 0.07) and highly rate the quality of care they received from them (p = 0.09).

**Table 2:** Ambulatory Care Experience Survey (ACES) Score Changes, by Experimental Group

	Control Physicians			Intervention Physicians			
Survey Measure	Pre-Intervention (n = 525)	Post-Intervention (n = 662)	Change	Pre-Intervention (n = 453)	Post-Intevention (n = 441)	Change	

**Quality of Physician-Patient Interactions**	85.6	85.9	0.3	85.8	88.7	**2.9**	**0.08**
Explains things	89.1	88.1	-0.9	88.2	91.3	**3.1**	**0.02**
Clear instructions	87.9	88.0	0.1	87.1	89.9	2.8	0.11
Listens carefully	86.8	87.1	0.3	86.8	88.6	1.9	0.18
Spend enough time	81.3	81.8	0.5	83.4	85.9	2.5	0.32
Knowledge of medical history	83.7	84.2	0.5	83.4	85.9	2.5	0.23
**Overall Rating of Physician**	84.6	84.8	0.2	84.0	86.2	**2.2**	**0.09**
**Willingness to Recommend Physician**	84.3	84.8	0.5	84.1	87.6	**3.5**	**0.07**
**Care Coordination**	72.3	71.9	-0.4	79.2	81.1	1.9	0.51
Follow-up on test results	68.8	67.8	-0.9	81.7	83.1	1.3	0.69
Informed about care	77.5	78.0	0.5	78.2	78.2	0.1	0.90
**Organizational Access**	80.5	77.8	-2.7	81.3	81.9	0.6	0.09
Timely sick care	84.8	81.5	-3.2	86.6	86.2	-0.4	0.04
Timely call back	79.2	75.0	-4.1	78.9	77.5	-1.4	0.21
After hours advice	68.2	72.3	4.1	74.5	74.7	0.2	0.71
**Office Staff Interactions**	83.0	82.2	-0.8	79.0	81.1	2.1	0.06

Note: Survey scores are adjusted for patient age, gender, education, race, self-rated physical health, sample derivation differences, and physician-patient relationship duration. Multilevel regression models that used random effects to account for the clustering of respondents within physicians were used to predict mean scores.

There was marginally significant differences in intervention and control physician performance on the organizational access summary measure (p = 0.09), but this was driven by declines in the performance of control group physicians over time. Patients' assessments of the care coordination (p = 0.51) and office staff interactions (p = 0.06) did not differ by experimental group.

### Physicians' Perspectives

During the follow-up group teleconferences, most physicians indicated that they were actively working to incorporate the communication and agenda-setting techniques in their practices. Experiences were diverse – some indicated that the techniques significantly enhanced their ability to conduct visits, while others believed that the techniques impeded their ability to complete visits in a timely manner.

Benefits attributed to the newly acquired agenda-setting skills were often as simple as *"being aware of verbal and non-verbal cues from the patient*", *"keeping quiet after asking a question"*, and *"trying to talk less at the beginning*". Contrary to some physicians' expectations that the introduction of new habits into the clinical encounter would involve disruptions or feelings of disorganization, some physicians reported actually "*feeling more in control*". One physician felt *"less stressed by simply knowing what was on the patient's mind"*, and was "*enjoying patient encounters more"*, suggesting that the benefits of the intervention are likely to enhance physician-patient relationship quality in the long run. Another physician reported *"feeling less rushed" *and *"feeling more in control by allowing the information [to] come" *to her. Using the facilitation techniques helped to *"bring out another aspect of the patient's problem that was very important to know, but only after the third 'uh-huh"'*.

In order to cope with obstacles such as running behind schedule as a result of trying to address all patient concerns, physicians used tools such as pre-visit questionnaires. These tools were generally quite helpful, but also had their own drawbacks. Some patients had symptom lists that were simply too long to address during a single visit. One physician remarked, *"When that pit of anxiety set in with the unraveling of the long list*, [she reminded herself] *to focus and prioritize"*, in order to ensure that the encounter was mutually beneficial. Some long-term users of the pre-visit questionnaire revealed that the "long symptom list" problem would resolve with time as patients became more familiar with the process.

Extra time spent during the clinical encounter was a common concern, and there were mixed reactions regarding the impact of integrating the agenda-setting skills into practice. Physicians expressed concerns with *"running late more often" *and that the intervention did "*not have any impact on timing in the case of young and healthy patients*". However, according to one physician, using the communication techniques during the clinical encounter *"takes more time now, but saves time later"*.

## Discussion

The findings from this agenda-setting intervention aimed at improving the quality of physician-patient relationships suggest that a simple and modest intervention (an evening workshop and two voluntary follow-up teleconferences with study participants) can have a positive impact on the quality of physician-patient interactions. In particular, intervention physicians improved their ability to explain things in a way that is easy for patients to understand. These results are encouraging about the potential for trainings of this sort to favorably influence the interaction skills of physicians in practice. Despite the modest magnitude of most changes, it is worth noting that a 3-point gain in scores has considerable practical significant for practices in the context of public reporting and pay-for-performance. In California and Massachusetts, for example, where statewide data on patient experiences with ambulatory practices have been publicly reported, a change of this magnitude would raise an organization's standings by about 40-points in percentile rank [28], e.g., from the 40th to 80th percentile or 50th to 90^th ^percentile. Moreover, our content analysis of the teleconferences suggests that the intervention resulted in significant changes in communication behavior. Time constraints associated with integrating the agenda-setting techniques into practice appeared to be a concern among some physicians. However, most physicians believed that striving for patient-centered communication and utilizing the agenda-setting skills would likely benefit their practice in the long run.

This study has some limitations. First, although intervention and control group physicians were matched using baseline survey scores, patient panel characteristics differed between the groups. However, we adjusted for a wide range of respondent characteristics to account for these differences. Second, due to differences in baseline and post-intervention sampling procedures, patients 65 years or older were excluded from the analysis. Our results might not generalize to older patients. Finally, the physicians were drawn from the lowest performing quartile among California physicians on the patient-reported experience quality indicators. As such, our results could only apply to poorer performing physicians. However, the finding that poorly performing physicians can improve their patient experience scores is significant and suggests the value of investing in skills training for this group.

## Conclusion

Our results suggest that a brief communication training intervention for practicing physicians with low patient survey scores has the potential to improve the quality of physician-patient interactions, as experienced and reported by patients. The relative success observed here may be due, in part, to a combination of the external pressures for high performance on these measures (e.g., pay-for-performance, public reporting) and the effect of providing the training to a group of physicians in practice together. Previous evidence has suggested that achieving improved communication requires considerably longer and more intensive training [24,30]. It will be important to evaluate the effect of more extensive trainings, including those that work with physicians on a broader set of communication techniques.

## Competing interests

The author(s) declare that they have no competing interests.

## Authors' contributions

Concept and design (DGS, RMF, WHR, TVG); collection of data (DGS); analysis and interpretation of data (HPR, MPA, EGO, WHR, DGS); drafting of the manuscript (HPR, EGO, DGS); critical revision of the manuscript for important intellectual content (HPR, MPA, RMF, TVG, WHR, DGS); statistical analysis (HPR, MPA); provision of study materials or patients (TVG); obtaining funding (DGS, TVG); supervision (HPR, DGS). All authors have read and approved the final manuscript.

## Pre-publication history

The pre-publication history for this paper can be accessed here:



## References

[B1] Finocchio LJ, Bailiff PJ, Grant RW, O'Neil EH (1995). Professional competencies in the changing health care system: physicians' views on the importance and adequacy of formal training in medical school. Acad Med.

[B2] Girgis A, Sanson-Fisher RW, McCarthy WH (1997). Communicating with patients: surgeons' perceptions of their skills and need for training. Aust N Z J Surg.

[B3] Girgis A, Sanson-Fisher RW, Walsh RA (2001). Preventive and other interactional skills of general practitioners, surgeons, and physicians: perceived competence and endorsement of postgraduate training. Prev Med.

[B4] Langille DB, Kaufman DM, Laidlaw TA, Sargeant J, MacLeod H (2001). Faculty attitudes towards medical communication and their perceptions of students' communication skills training at Dalhousie University. Med Educ.

[B5] Markakis KM, Beckman HB, Suchman AL, Frankel RM (2000). The path to professionalism: cultivating humanistic values and attitudes in residency training. Acad Med.

[B6] Sise MJ, Sise CB, Sack DI, Goerhing M (2006). Surgeons' attitudes about communicating with patients and their families. Curr Surg.

[B7] Safran DG, Taira DA, Rogers WH, Kosinski M, Ware JE, Tarlov AR (1998). Linking primary care performance to outcomes of care. JFamPract.

[B8] Stewart M, Brown JB, Donner A, McWhinney IR, Oates J, Weston WW, Jordan J (2000). The impact of patient-centered care on outcomes. JFamPract.

[B9] Federman AD, Cook EF, Phillips RS, Puopolo AL, Haas JS, Brennan TA, Burstin HR (2001). Intention to discontinue care among primary care patients: influence of physician behavior and process of care. JGenInternMed.

[B10] Keating NL, Green DC, Kao AC, Gazmararian JA, Wu VY, Cleary PD (2002). How are patients' specific ambulatory care experiences related to trust, satisfaction, and considering changing physicians?. JGenInternMed.

[B11] Safran DG, Montgomery JE, Chang H, Murphy J, Rogers WH (2001). Switching doctors: predictors of voluntary disenrollment from a primary physician's practice. JFamPract.

[B12] Montgomery JE, Irish JT, Wilson IB, Chang H, Li AC, Rogers WH, Safran DG (2004). Primary care experiences of medicare beneficiaries, 1998 to 2000. JGenInternMed.

[B13] Murphy J, Chang H, Montgomery JE, Rogers WH, Safran DG (2001). The quality of physician-patient relationships. Patients' experiences 1996-1999. J Fam Pract.

[B14] Stein T, Frankel RM, Krupat E (2005). Enhancing clinician communication skills in a large healthcare organization: a longitudinal case study. Patient Educ Couns.

[B15] Stein TS, Kwan J (1999). Thriving in a busy practice: physician-patient communication training. Eff Clin Pract.

[B16] Baile WF, Kudelka AP, Beale EA, Glober GA, Myers EG, Greisinger AJ, Bast RC, Goldstein MG, Novack D, Lenzi R (1999). Communication skills training in oncology. Description and preliminary outcomes of workshops on breaking bad news and managing patient reactions to illness. Cancer.

[B17] Chan CS, Wun YT, Cheung A, Dickinson JA, Chan KW, Lee HC, Yung YM (2003). Communication skill of general practitioners: any room for improvement? How much can it be improved?. Med Educ.

[B18] Roter DL, Larson S, Shinitzky H, Chernoff R, Serwint JR, Adamo G, Wissow L (2004). Use of an innovative video feedback technique to enhance communication skills training. Med Educ.

[B19] Sliwa JA, Makoul G, Betts H (2002). Rehabilitation-specific communication skills training: improving the physician-patient relationship. Am J Phys Med Rehabil.

[B20] Cornuz J, Humair JP, Seematter L, Stoianov R, van Melle G, Stalder H, Pecoud A (2002). Efficacy of resident training in smoking cessation: a randomized, controlled trial of a program based on application of behavioral theory and practice with standardized patients. Ann Intern Med.

[B21] Fallowfield L, Jenkins V, Farewell V, Solis-Trapala I (2003). Enduring impact of communication skills training: results of a 12-month follow-up. Br J Cancer.

[B22] Moral RR, Alamo MM, Jurado MA, de Torres LP (2001). Effectiveness of a learner-centred training programme for primary care physicians in using a patient-centred consultation style. Fam Pract.

[B23] Roter DL, Hall JA, Kern DE, Barker LR, Cole KA, Roca RP (1995). Improving physicians' interviewing skills and reducing patients' emotional distress. A randomized clinical trial. ArchInternMed.

[B24] Brown JB, Boles M, Mullooly JP, Levinson W (1999). Effect of clinician communication skills training on patient satisfaction. A randomized, controlled trial. AnnInternMed.

[B25] Krupat E, Frankel R, Stein T, Irish J (2006). The Four Habits Coding Scheme: validation of an instrument to assess clinicians' communication behavior. Patient Educ Couns.

[B26] Beckman HB, Frankel RM (1984). The effect of physician behavior on the collection of data. Ann Intern Med.

[B27] Rost K, Frankel R (1993). The introduction of the older patient's problems in the medical visit. J Aging Health.

[B28] Safran DG, Karp M, Coltin K, Chang H, Li A, Ogren J, Rogers WH (2006). Measuring patients' experiences with individual primary care physicians. Results of a statewide demonstration project. JGenInternMed.

[B29] Rodriguez HP, von Glahn T, Chang H, Rogers WH, Safran DG (2007). Patient Samples for Measuring Primary Care Physician Performance: Who Should Be Included?. Med Care.

[B30] Smith RC, Lyles JS, Mettler J, Stoffelmayr BE, Van Egeren LF, Marshall AA, Gardiner JC, Maduschke KM, Stanley JM, Osborn GG, Shebroe V, Greenbaum RB (1998). The effectiveness of intensive training for residents in interviewing. A randomized, controlled study. Ann Intern Med.

